# Complex Floral Scent Profile of *Neottia ovata* (Orchidaceae): General Attractants and Beyond

**DOI:** 10.3390/plants14060942

**Published:** 2025-03-17

**Authors:** Edyta Jermakowicz, Marcin Stocki, Piotr Szefer, Justyna Burzyńska, Emilia Brzosko

**Affiliations:** 1 Department of Plant Biology and Ecology, Faculty of Biology, University of Bialystok, 15-245 Białystok, Poland; j.burzynska@uwb.edu.pl (J.B.); emilka@uwb.edu.pl (E.B.); 2 Faculty of Civil Engineering and Environmental Sciences, Institute of Forest Sciences, Białystok University of Technology, 15-351 Białystok, Poland; m.stocki@pb.edu.pl; 3 Faculty of Science, University of South Bohemia, České Budějovice, Branišovská 1645/31a, 370 05 České Budějovice, Czech Republic; szefer85@gmail.com; 4 Biology Centre, Institute of Entomology, Czech Academy of Science, Branišovská 31, 370 05 České Budějovice, Czech Republic

**Keywords:** GC–MS, SPME, generalized pollination strategy, terpenes, orchid pollination, flower volatiles, pollinator guilds

## Abstract

Understanding the complexity of flower scent—a crucial attractant for pollinators and a key factor in ensuring plant reproduction—is an essential ecological task for highly endangered orchids. To address this issue, we studied the flower volatiles profile of *Neottia ovata*, a nectar-rewarding orchid known for its generalist pollination strategy. We then compared the chemical composition of *N. ovata* floral scent with scent data of other orchid species to place our findings in the context of general volatile attractants emitted by nectar-rewarding or food-deceptive species. Our results contribute to understanding the complexity of the *N. ovata* floral scent profile and provide valuable methodological insights. The scented bouquet of *N. ovata* comprises 100 compounds with a relatively consistent composition across the analyzed samples. It is rich in terpenes, including linalool and trans-/cis-sabinene hydrate, compounds commonly associated with generalized rewarding or food-deceptive pollination systems. Other terpenes identified include α- and β-pinene, limonene, and β-phellandrene, whose presence underscores the generalized nature of the floral scent. Interestingly, in the studied *N. ovata* populations, the dominance among terpenes is shifting markedly towards γ-terpinene, α-terpinene, and terpinene-4-ol, commonly found in essential oils and the floral scents of some supergeneralist-pollination plants. Aromatic compounds were less represented in the *N. ovata* scent profile and those of other orchids studied, though benzyl alcohol and benzaldehyde were noticeably more abundant. Aliphatic compounds composed the least prevalent fraction, showing a marked decreasing trend among nectar-rewarding species with generalized or specialized pollination systems. It is worth emphasizing that the applied methodology revealed an extensive group of low-frequency compounds in the *N. ovata* floral scent. This finding raises new ecological questions about the intraspecific diversity of floral scent profiles and sheds new light on the factors determining effective reproduction in this species of orchid.

## 1. Introduction

Orchids are renowned for their diverse pollination systems, which involve a variety of attraction mechanisms, including floral scents [1]. As the most complex floral signal, scent is undoubtedly a trait of significant importance in plants’ adaptive potential within intricate pollinators’ networks [2,3,4,5,6].

Floral scents are mixtures of organic compounds that vary in chemical properties, molecular weight, polarity, and oxidation state, produced through anabolic and catabolic processes of various biosynthetic pathways [7,8]. These floral volatiles can be categorized into three main groups: terpenoids, phenylpropanoids/benzenoids (aromatic compounds), and fatty acid derivatives (aliphatic compounds) [9]. Approximately 1700 volatile compounds emitted by flowers have been identified till now, with about 50% originating from orchid taxa [10,11]. Recent advancements in analytical methods have significantly improved techniques for capturing volatiles, enhancing both the sensitivity of research and the reproducibility of analyses. These developments have accelerated the growth of knowledge about floral scent chemistry and provided new insights into the immense complexity of volatile biochemical signals [9,12,13,14]. An ever-expanding body of data confirms the substantial variation in this floral trait, with notable differences in scent composition even within the same species [15,16,17,18]. Studies have documented that the quality and quantity of final scent composition are influenced by biotic and abiotic environmental factors [18,19,20,21,22]. Among these factors, the pollination system is one of the most significant drivers of scent diversification [21,23]. Pollination systems in orchids, a model group for studying plant–pollinator coevolution, are renowned for their specialization toward specific pollinator groups, primarily due to distinct floral morphology (e.g., the shape of the corolla or the structure of the gynostemium). In contrast, generalist pollination, an ancestral system among plants, typically involves multiple functional groups of pollinators [24]. Orchids that occupy the generalist end of the generalization–specialization continuum of pollination systems are relatively rare [25,26,27,28,29,30,31]. These orchids often have phenotypically specialized flowers that can be pollinated by a wide range of floral visitors, varying in size, mouthparts, and food preferences [16].

In this context, the scent profiles of generalist plants tend to be more complex than those of plants with specialist pollination systems, as they must appeal to the preferences of diverse insect species. Some general scent attractants, particularly those associated with nectar feeding, are present in food-rewarding and food-deceptive orchid species—two common pollination strategies among orchids [32]. However, due to stabilizing selection, the scent bouquets of food-rewarding species appear less variable than those of species employing deceptive pollination strategies [33].

Functionally specialized flowers with general attractants are known in various orchid taxa. One example is *Neottia ovata* (L.) Bluff & Fingerh., which has open nectaries and accessible nectar, adapted to the exploratory behavior of insects from distinct functional groups. Its floral traits, such as the lip adapted for landing and feeding insects and a longitudinal groove containing accessible nectar, facilitate these interactions [34]. On the other hand, the sepals and petals of *N. ovata* form a hood-like structure around the gynostemium, with nectar accumulating at the base of the lip. This configuration might be considered a specialized floral adaptation [35]. *Neottia ovata* produces several dozen yellow-greenish flowers per inflorescence. Despite this, the plant is inconspicuous and poorly visible within the surrounding greenery. Consequently, olfactory attraction is central in drawing a broad spectrum of *N. ovata* pollinators [26,34]. Nilsson [26] reported that nearly 300 insect species visit *N. ovata* flowers, with 50 described as the most efficient due to the frequent presence of pollinia on their bodies. Among Swedish populations, ichneumonids, particularly males, were the most frequent pollinators [26]. In other regions, such as Spain and Belgium (Claessens and Kleynen [34]), Great Britain [36], and Poland (unpublished observations), Coleoptera and Ichneumonidae are commonly observed visitors. However, the range of visiting insect species is broader. This variety of visiting insects, with distinct sensory perceptions and ecological requirements, likely relies on nectar as the primary attractant. However, additional luring mechanisms, including complex blends of volatile compounds, also play a crucial role. General volatiles likely serve as long-distance attractants, while other scent components, such as sexual or aggregation pheromones, may specifically target the most reliable pollinators [37]. Understanding the adaptive potential of generalist pollination species, like *N. ovata*, to interact with complex pollinator communities is of great importance in the context of ongoing climatic and environmental changes to which insects are exceptionally responsive [38].

Thus, our research aimed to (i) obtain a comprehensive profile of the chemical composition of *N. ovata* floral scent and (ii) identify both general scent components and those with potentially specific functions. To achieve this, we analyzed the flower scent of *N. ovata* from various populations within the center of its distribution range. By comparing it with other orchid species, we identified common, general components and rare, potentially species-specific elements in the flower scent of *N. ovata*.

## 2. Material and Methods

### 2.1. The Floral Scent of Neottia ovata—Sampling and Analysis

The plant material was collected in three populations of *N. ovata* localized in northeast Poland. One is in Knyszyńska Forest (KL), and two are in Suwałki Landscape Park (TW and TR). *Neottia ovata* is a rare species under legal protection in Poland; thus, the material was collected with special permissions (WPN.6400.51.2020.MK). All samples were collected in optimum flowering in June 2020. The KL population is connected with the anthropogenic habitat, within a plant community dominated by grasses and sedges, under a canopy of shrubs–trees (*Salix* spp., *Alnus* spp.). The TW and TR populations’ habitats are shaded places within alder forests with dense, multi-species undergrowth, while the TW population is localized on the bog fed by shallow springs.

Six *N. ovata* inflorescences, containing a minimum of 10–15 opened flowers each, were enclosed in 60 mL headspace vials, then transported to a laboratory at the Institute of Forest Sciences, Białystok University of Technology (Poland), and underwent chemical research on the same day. The volatile compounds emitted from *N. ovata* inflorescences were analyzed by headspace solid-phase microextraction and gas chromatography with mass spectrometry (HS-SPME/GC–MS). The inflorescence of *N. ovata* was placed in a headspace vial, closed with a cap with a septum, and was heated for 30 min at 30 °C. Afterwards, the septum was pierced, and SPME fiber with the divinylbenzene/carboxy/polydimethylsiloxane (DVB/CAR/PDMS) stationary phase was exposed in the headspace gas phase for 30 min. Next, the SPME fiber was introduced for 10 min into the GC–MS injection port. The GC–MS analyses were performed using an Agilent 7890A gas chromatograph coupled with an Agilent 5975C mass spectrometer. The injector worked in a splitless mode at a temperature of 250 °C. Chromatographic separation was conducted on an HP-5MS capillary column (30 m × 0.25 m × 0.25 μm) with a 1 mL/min helium flow rate. The starting column temperature was 35 °C, and this was increased to 250 °C at a rate of 5 °C/min. The ion source and quadrupole temperature were 230 °C and 150 °C, respectively. The ionization energy in the mass spectrometer equaled 70 eV. The detection was performed in full scan mode for 29–600 units. After peak integration, the percentage content of compounds in the total ion current (%TIC) was calculated. For the identification of volatiles, both mass spectral data and retention indices were used. Mass spectrometric analysis was done using NIST [39] and Wiley [40] mass spectral libraries, as well as Adams [41] and Tkachev [42] collections. The retention indices of the volatile compounds were calculated by considering the *n*-alkane retention times.

### 2.2. General and Specific Volatile Constituents—Reference Data Selection and Analyses

We reviewed the available literature data to compare the scent chemical composition of *N. ovata* with the flower scent of other orchid species similar in terms of pollination systems. We selected orchids of generalized and specialized pollination systems, both nectar-rewarding and general food-deceptive, of which we can assume the similarity of general volatile flower attractants that they use. To describe the pollination system as generalized or specialist, we search for orchid species for which pollinator observations were made. We also limited our search to terrestrial species from higher latitudes (with some exceptions). The available literature was reviewed using Google Scholar, Web of Science, and Research Gate platforms. The first step of searching revealed that many publications give lists of chemical compounds in the orchid flower fragrances. However, they often lack data concerning pollination syndrome, with no observations of flower visitors and effective pollinators. Thus, the available publications were thoroughly checked, and those with the required data were chosen. The final list consisted of 23 orchid species. Data included in the analysis derived from single or multiple populations; the data were treated as species properties (without considering intraspecific variability). Chemical compounds of the flower scent, constituting 1% in at least one of the analyzed scent samples, were considered.

In the case of the considered orchids, the method for analyzing volatile compounds emitted by the flowers was constant; however, the techniques for collecting and extracting these compounds differed (solvent extracts, microextraction, thermal desorption, distillation–extraction method) (Table S1), which undoubtedly influenced the scope of the extracted compounds. Despite the differences in extraction methods used, a certain repetitiveness of compounds was noticeable, which we analyzed in terms of their occurrence in particular orchid pollination groups. Thus, we conducted analyses to discern similarities in compounds’ presence in the flower scent among specialist (S) and generalist (G), as well as between food-deceptive (FD) and nectar-rewarding (RW) orchid species, that putatively used the same flower volatile attractants. Additionally, to direct the analysis, we explored the relationship along the combined pollination system groups: nectar-rewarding generalist (G_RW), food-deceptive generalist (G_FD), as well as nectar-rewarding specialist (S_RW) and food-deceptive specialist (S_FD). The chemical compounds were analyzed in groups: terpenes, aliphatic, and aromatic compounds. These analyses were executed using the mdatools library in R (R Core Team 2023, v. 4.3.2) [43]. We replicated the aforementioned analyses using a reduced dataset, wherein chemical compounds found exclusively in a single species were excluded. This removal did not substantively alter the results. Thus, for easy visual comparison, a set of heatmaps was generated using a complete dataset, and a dendrogram was constructed using Euclidean distance as a similarity measure. The ComplexHeatmap library in R [44] was used for that purpose.

## 3. Results

### 3.1. Floral Volatiles of Neottia ovata

The flowers of *N. ovata* collected from the natural populations from northeast Poland emitted a complex mixture of volatiles. A total of 100 volatile compounds were detected, from which 99 were identified (Table 1). The most frequent group in the *N. ovata* volatile composition were terpenoids, particularly monoterpenes (42.91–81.64%). Of the remaining groups, the largest were aliphatic alcohols (4.60–23.68%) and aliphatic aldehydes (3.03–15.72%). The group of compounds that exceeded 10% were monoterpenes, in particular: terpinen-4-ol with 7.12–36.74% in different samples, γ-terpinene with 6.63–16.98%, and linalool, accounting for 1.55–12.24%. The medium-account compounds (4–10%) also included monoterpenes—α-terpinene, cis- and trans-sabinene hydrate, α-terpinolene—as well as aliphatic aldehydes, including isopentanal, octanal, and nonanal, and aliphatic alcohols: methanol, ethanol, and 1-octanol. Medium-account compounds were also found among alkanes and alkenes, and in aromatic compounds benzeneacetaldehyde and 2-phenylethanol (Table 1). The obtained chromatograms mostly overlap; about 50 compounds were present in all samples, and 28 compounds were found only once in a single sample, mostly in a low amount (<1% TIC) (Table 1). Certain differences are noticeable between samples from different populations, while the TR population stands out here, with a higher amount of sesquiterpenes, aliphatic acids, and alkanes and alkenes, with an absence of ketones (Table 1). However, we consider this aspect a basis for further, in-depth interpopulation analyses due to the insufficient sample size.

### 3.2. General and Specific Floral Volatiles of Rewarding and Food-Deceptive Orchids

**Figure 1:**
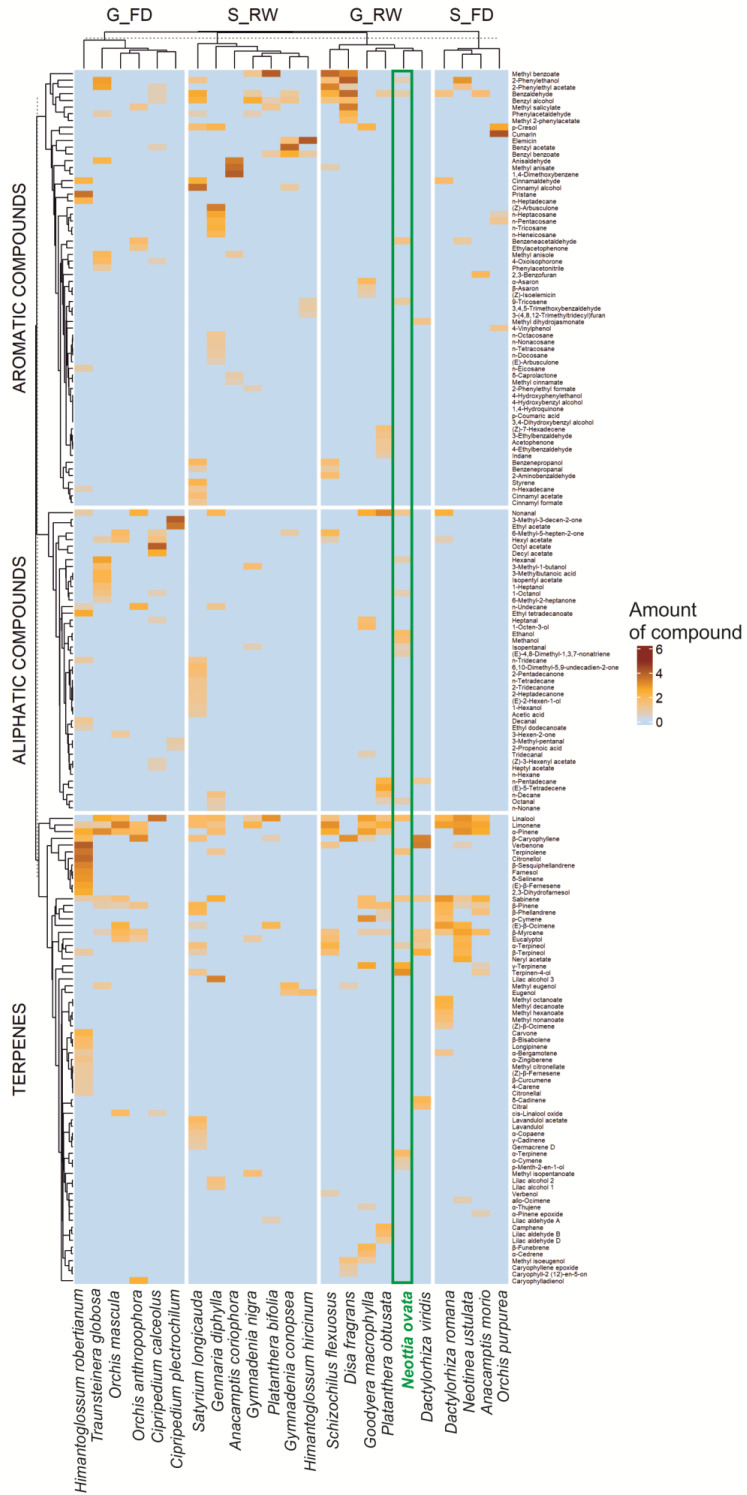
Occurrence and relative amount of 180 flower scent compounds (terpenes, aliphatic com-pounds, aromatic compounds) found in 23 orchid species, grouped according to pollination strategies (FD—general food-deceptive, RW—nectar-rewarding, G—generalist, S—specialist pollination) visualized by heatmap; amount of compound—amount of a given compound in relation to the entire sample (transformed value). For details on the collected data, see Table S1.

We analyzed the scent compositions of 23 orchid species (Table S1), including 180 chemical compounds (after review and unification of chemical nomenclature). They were classified into three main groups (terpenes, aliphatic, and aromatic compounds). The comparison of *N. ovata* to other generalist-rewarding orchids indicates a high content of terpenes but also highlights the uniqueness of *N. ovata*’s scent, with its exceptionally high levels of γ-terpinene, terpinen-4-ol, α-terpinolene, and aliphatic alcohols, such as ethanol and methanol (Table 1, Figure 1).

Despite different methods used to study the floral scent (extraction methods), the analyzed orchids’ volatile profiles revealed that 50 chemical compounds occurred in more than one species (Figure 1). In contrast, all others were found in a single species (Table S2). In summary, across the terpenes, aliphatic compounds, and aromatic compounds, there were 52 (70 total), 26 (45), and 43 (65) single compounds, respectively (Table S2). The most common compounds across all groups were terpenes. Aromatic compounds occurred less frequently, while aliphatic compounds were the least common. There was also a slightly higher proportion of aromatic compounds among rewarding orchid species, both visited by comprehensive (generalized pollination system) and narrow groups (specialized pollination system) of potential and most effective pollinators (Figure 1).

## 4. Discussion

Our results document that the floral scent bouquet of the generalist-pollination-strategy orchid *Neottia ovata* is more complex than previously reported. We found 100 floral volatile compounds, of which 99 were identified, with monoterpenes, aliphatic aldehydes, and aliphatic alcohols being the most abundant. Nilsson [26] found 24 chemical compounds in the Swedish population of this species, of which 17 were identified, with over 90% being monoterpenes and sesquiterpenes, among which linalool and trans-β-ocimene were the most abundant. The recent work of D’Auria et al. [45] on the *N. ovata* flower scent documents 21 volatile compounds, mainly alkanes such as heptadecane and pentadecane. Beyond intraspecific differences, those considerable variances in the scent composition between cited and presented data may result from methodological differences. Nilsson [26] extracted compounds from adsorbents with pentan and diethyl ether using the adsorption–extraction collecting method and GC–MS technique. D’Auria et al. [45] used SPME fibers with the DVB/CAR/PDMS stationary phase, as we did. However, the exposition time in the headspace gas phase differed, being 24 h in their case, in contrast to the 30 min exposition time we used. Referring to these results, Tholl et al. [14] emphasize that controlling sampling parameters, e.g., exposition time, is crucial for optimal analytical results. Specifically, the same authors define the equilibration between the SPME fiber and the volatile sample as lasting a few minutes to half an hour, depending on the compound’s volatility and polarity and the adsorbent’s properties. As mentioned above, among the identified floral volatiles, monoterpenes, aliphatic aldehydes, and alcohols were present in the highest proportions, while the content of alkanes did not exceed 10% of the TIC. This contrasts with the work of D’Auria et al. [45], where alkanes constituted the dominant fraction.

Among the reviewed orchids, using the SPME technique to adsorb floral volatile compounds was still rare. However, an increase in the richness of compounds in the profiles obtained using this method is noticeable.

Besides the methodology used, various environmental factors (geography, habitat, climate) influence flower scent composition [19,20,21,22,47]. Despite the methodological differences, the available data on the scent of *N. ovata* flowers, gathered from several sources, provide a comprehensive and nuanced picture of the volatile blends emitted by this species’ flowers. With certainty, improving the sampling methods, especially connected with headspace sampling that captures volatile compounds from the air surrounding flowers, was the breakthrough [9,14,48]. Volatiles are enriched on the sorbent covering SPME fiber, which allows the detection of even low-abundance compounds from low-emitting flowers, of which *N. ovata* is a type [14]. The low availability of orchid flowers and the low abundance of semiochemicals are two of the most critical limitations in analyzing orchid fragrances.

The spectrum of volatile compounds emitted by *N. ovata* flowers creates a complex scent profile. It stands out with a relatively diverse pool of compounds and constant biochemical composition. The complex biochemical composition is resolved as an adaptation to a diverse group of potential pollinators [49]. Meanwhile, relative homogeneity acts as an advertisement for a high-quality reward or a reward with unique properties (proportions of sugars and amino acids) that make such a source desirable to flower visitors [50]. These features refer to a generalized pollination system and reproductive assurance strategy within a variable and unpredictable pollinator community [51,52]. The scent of *N. ovata* is described as sweet and related to aromatic constituents of the nectar, which is rich in glucose, fructose, and amino acids [30].

Moreover, the importance of scent as an attractant is also emphasized by the anatomical characteristics of flowers closely related to the emission of volatile compounds [35]. We did not investigate the intraspecific variability of the *N. ovata* flower scent, while the profiles of individuals deriving from three different localities showed repeatability. Almost 70% of compounds appear in more than two analyzed samples, and the components that most differentiate the samples occur with the lowest abundance (<1% TIC).

Regardless of the method used, when we considered flower volatiles of orchid species, both the nectar-rewarding and food-deceptive, the generalist and specialist ones, about 10% of components occurred frequently (in 5–10 of the species). Approximately 50–60% of compounds were found only in the scent of a single species, which can be linked both to the specificity of the species and the extraction method used. The most frequently recurring floral scent compounds are terpenes and aromatic compounds (benzenoids), known for their biological activity and as general attractants that initiate a response in various flower visitors [9,10]. It should be emphasized that the floral scent is a blend of components with synergistic effects, of which we still know very little. Only the behavioral and electrophysiological experiments (EAD) allow for evaluating the biological activity of specific flower fragrance components. However, they are still limited, in orchids, primarily to sexually deceptive pollination systems, where the scent profile is often poor, with one or two active components [53,54,55].

Within those universal, general scent attractants, terpenes are among the most important groups, primary in character, associated with repelling floriferous animals and antibacterial and antifungal activity [56]. From this biochemical group, linalool is one of the most abundant components of the *N. ovata* scent bouquet, as reported by Nillson [26], and also the most essential and repetitive general scent attractant, luring different groups of pollinators [9]. This acyclic monoterpene has been confirmed to be active as a long-distance attractant and is connected with the fragrance emitted by nectar [50,57]. Linalool was also considered in pollination systems, where bee pollinators search for a perfume reward, but rather as a long-distance decoy [58]. In turn, Burdon et al. [50] reveal that *Bombus impatiens* could detect the differences in linalool emission by flowers and choose those with a higher rate. In general, the quality of scent, including terpene concentration—mainly linalool, limonene, and β-pinene—serves as a signal for pollinators to identify the floral stage and presence of nectar [56,59]. Simultaneously, linalool is almost absent in the fragrance composition of orchid flowers that exhibit specialized pollination systems [60], which may significantly limit the number of insect visits, especially of general pollinators.

Another monoterpene found among compared orchids, with a general scent character, is α-pinene, one of the significant aphid alarm pheromones that attract hoverfly females [61]. The monoterpenes α-pinene and β-pinene, with appropriately huge concentrations, have elicited a response to flowers from *Bombus terrestris* [62]. Another bicyclic monoterpene, sabinene, is found among flower volatiles of *N. ovata* and in almost half of the analyzed orchids’ flower scent profiles. This compound contributes to spicy, citrusy, and piney aromas that are detectable by the human senses. Furthermore, among orchids, sabinene is found in euglossophilous orchid flowers, which together with pinene, limonene, and α-terpineol forms the final fragrance combination, which is attractive to bees searching for perfume reward [60]. Activity connected with insect pheromones was also defined for myrcene and β-phellandrene [61], commonly found components of different orchid flower scents. All the mentioned terpenes are found in the scent profiles of the considered orchid species that lure with the promise of real or false reward, although in the flower scent of *N. ovata*, dominance visibly shifted towards γ-terpinene, α-terpinene, and terpinen-4-ol. These terpenes were reported with high concentrations in the essential oils and flower scent of supergeneralist pollination species from the Apiaceae family, specifically in the flower scent of *Aegopadium podagraria*, *Anthriscus sylvestris*, and *Heracleum sibiricum* [63]. Zito et al. [64] noted the dominance of terpinolene and γ-terpinene, combined with linalool in some fly-pollinated plants, where this mixture constituted a “sweet scent” that elicited the response of sapromyophilus houseflies. γ-terpinene is also a dominant component in thyme essential oil, which is, in this case, strictly linked with antioxidant and antibacterial properties [65]. The vital role of terpene alcohols, including α-terpineol, terpinene-4-ol, and linalool, in the antagonistic relationships of plants with other organisms is often emphasized [56]. However, in the case of the Orchidaceae family, these compounds appear to gain a contrary function, luring the potential pollinators [9]. Terpinen-4-ol is, for example, one of the dominant compounds in the intensely floral fragrant flowers of the tropical orchids *Prosthechea vespa* and *P. fragrans* [66].

Known for attracting to its flowers many taxonomically disparate insect visitors are the species of umbellifer *Angelica sylvestris*, with the domination of α-pinen, β-phellandrene [67] and β-myrcene, benzaldehyde, and phenylethanol in the flower volatiles [68]. Those two last aromatics, together with benzeneacetaldehyde, benzyl alcohol, and methyl benzoate ester, were iterative components in the flower scent profiles of nectar-rewarding and food-deceptive orchids considered here. Relatively rich pools of aromatic compounds were present in the analyzed orchid scent profiles. The dominant compounds varied, but those mentioned above appeared with noticeably higher frequency and often in a significant proportion within the profile, which can also indicate their role as general attractants. The aromatic ester methyl benzoate, found in generalized-pollination, nectar-rewarding species like *Disa fragrans*, *Schizochilus flexuous*, and *Dactylorhiza viridis*, is also emphasized as a compound connected with the scent of nectar [50], causing EAD activity in pollinators of several orchids [69]. Also among iterative components in the flower scent of nectar-rewarding and food-deceptive orchids were aromatic alcohols, such as benzyl alcohol, 2-phenylethanol, and aromatic aldehydes, mainly benzeneacetaldehyde and benzaldehyde. The last three we found in the *N. ovata* scent as not dominant compounds, but constant ones.

The aliphatic compounds were the least represented floral volatile group in the profiles of the studied orchids. Aliphatics, specifically alkanes and alkenes, play a vital role in the pollination of sexually deceptive orchids, and in such cases, they can dominate or be the scarcer elements of the fragrance composition [70]. In the analyzed group of orchids, the more frequent occurrence of two aliphatic compounds, specifically aldehyde nonanal and ketone 6-methyl-5-hepten-2-one, is noticeable. Nonanal is found in the scent of *N. ovata* in small quantities. In contrast, the flower scent of other orchids, such as *Platanthera obtusata* or *Malaxis monophyllos*, is nonanal-rich, which is strongly connected with luring mosquitos as pollinators [71,72]. A similar activity attracting mosquitoes is reported for 6-methyl-5-hepten-2-one [9], which was also noted in the flower scent of sexually deceptive *Ophrys insectifera* [73].

The highly sensitive SPME method, which is a selective method, provided us with a compound-rich, complex picture of the scent profile of *N. ovata* flowers, which might be still incomplete. Our study reveals an extensive group of low-frequency compounds, which occurring with an average rate of around 1% of the total volatiles. Unique compositions of low-frequency, specific scent compounds may show chemical similarities to, for example, compounds involved in insect chemical communication systems connected with activities other than feeding insects’ behavior, e.g., sexual/courtship activities or brood-site seeking. They may be directed towards a narrow group of the most effective pollinators, standing out from a large group of general visitors, or they may function only in populations where the selective pressure of a particular group of pollinators takes place [38]. Thus, our findings provide a basis for further, detailed experiments.

*Neottia ovata*, as a rewarding species with freely accessible nectar, is visited by a wide variety of insects: Hymenoptera (mostly sawflies), Diptera, and Coleoptera [34]. Among those, the ichneumonid wasps proved to be the most efficient group in some populations [26]. Generally, wasps were given as pollinators for different orchid species, employing various pollination strategies [34,70]. Recently, co-evolutionary systems utilizing sexual deception have been frequently analyzed in species from the genera *Chiloglottis*, *Drakaea*, and *Caladenia* with thynnine wasps as pollinators [74]. The scent of these orchid species corresponds to wasps’ female sexual pheromones and is dominated by compounds from the chiloglottones group and nitrogenous pyrazines [60]. Similarly, in the case of some *Ophrys* species (*O. speculum*), which are pollinated by wasps from the Scoliidae and Sphecidae families, they are attracted by the enantiomeric composition of 9-hydroxydecanoic and 7-hydroxyoctanoic acids (aliphatic, hydroxy-carboxylic acids), which also mimic female sex pheromones [75]. Ichneumonidae is an immensely vast group (>50,000 species) of parasitic wasps that utilize other insects (beetles, other wasps, bees, butterflies) as hosts [76]. Although the free-living adults feed on nectar and pollen, the knowledge regarding the significance of this group as pollinators is limited. Ichneumonid wasps are known for their involvement in nectar-rewarding or food-deceptive pollination strategies, but the best-documented within this group of insects are sexually deceptive systems. An example is a sexually deceptive *Cryptostylis* orchid [77], which is well-researched regarding semiochemicals used as volatile attractants. To achieve pollination, *Cryptostylis ovata*, for example, uses oxygenated tetrahydrofuran derivatives as semiochemicals that attract males of ichneumonid wasps *Lissopimpla excels* [78]. It is important to emphasize that the furan derivatives, specifically 2-ethyl furan, 2-pentylfuran, and (E)-dendrolasin, are present in the *N. ovata* flower scent. Other orchid species, which we compared, that lure Ichneumonidae as pollinators are *Dactylorhiza viridis* and *Chamaeorchis albida*, both nectarous with a honey-like scent [34,79]. Ichneumonid wasps as pollen vectors were also observed in terms of *Orchis anthropophora* [34], a food-deceptive species, in which the volatile components of flower scent strongly resemble those of nectar-rewarding species, due to the presence of terpenoids such as α-pinene, eucalyptol, caryophyllene, and aliphatic aldehyde nonanal [80].

## 5. Conclusions

*Neottia ovata* produces a complex floral scent with a high concentration of volatiles from groups commonly emitted by flowers of other generalized-pollination orchids, including orchids that lure pollinators by nectar reward and deceive them by the promise of food. On the other hand, a blend of additional, peculiar components may decrease competition with other co-flowering plants and reduce pollen loss by more efficiently luring a limited pool of the most efficient pollinators.

Moreover, if we accept that it is possible to speculate about the pollination syndrome by looking at the suite of floral traits [81], then we could ask how informative the data concerning scent composition are in the context of luring groups of pollinators and pollination syndrome. This can be especially essential regarding species with rare, hard-to-observe pollinators, a prevalent situation in the Orchidaceae family. In such cases, the volatile composition itself provides valuable clues. It would assist in establishing pollination strategies and narrowing down the search for successful pollinators, if they still need to be discovered. This approach, however, requires greater consistency and a standardized methodology.

Variation in fragrance chemistry is a prerequisite for scent-driven floral evolution, and becomes particularly important in the face of ongoing changes in habitats and pollinator communities. There have been frequently formulated questions regarding the extent of scent plasticity and its modification in response to changes in pollinator selection pressure, especially for specific pollination systems. The generalized pollination system adapts to an inconsistent pollinator community, but what if one of the insect groups, and consequently one of the selective pressures, disappears? Could the broad spectrum of floral volatiles also decrease, or will it act as insurance against the negative impacts of pollinator changes for plant species? To answer these questions, adopting a broad approach that includes multiple populations over an extended period, the most sensitive methods for capturing and analyzing scent samples, and detailed observations of visiting insects is essential.

## Figures and Tables

**Table 1:** A list of volatile compounds emitted by *N. ovata* flowers, obtained using the SPME/GC–MS method—the presented results (NT) with summary of the available literature data [26,45]. Number CAS—Chemical Abstracts Service number; t_ret._—retention time; RI_exp._—experimental retention index; RI_lit._—retention index from the literature (^a^—NIST [39]; ^b^—Adams [41]; ^c^—Tkachev [42]; ^d^—Isidorov [46]; N/A**—not available); TIC—total ion current; tr—trace; <0.1; ?—uncertain data.

					NT (n = 6)	Nillson (1981) [26]	D’Auria, et al. (2024) [45]
Compound	Number CAS	t_ret._ (min.)	RI_exp._	RI_lit._	TIC (%) Mean (min.–max.)	Presence (+) Absence (−)	Presence (+) Absence (−)
**Monoterpenes**					**61.5 (42.91–81.64)**		
α-Thujene	2867-05-2	7.910	922	924 ^b^	0.5 (tr-0.96)	**−**	**−**
α-Pinene	80-56-8	8.093	930	932 ^b^	0.71 (0.1–2.65)	**−**	**−**
Sabinene	3387-41-5	9.242	968	969 ^b^	0.6 (0.3–1.48)	**−**	**−**
β-Pinene	127-91-3	9.332	972	974 ^b^	0.52 (0–0.6)	**−**	**−**
α-Phellandrene	99-83-2	10.152	1002	1002 ^b^	0.63 (0–0.73)	**−**	**−**
δ-3-Carene	13466-78-9	10.317	1008	1010 ^c^	0.71 (0.29–1.29)	**+**	**−**
Myrcene					-	**+**	**−**
α-Terpinene	99-86-5	10.515	1015	1017 ^c^	5.44 (3.08–7.79)	**+**	**−**
o-Cymene	527-84-4	10.744	1022	1022 ^b^	1.42 (0.82–2.03)	**−**	**−**
Limonene	138-86-3	10.874	1027	1027 ^d^	0.94 (0–1.14)	**+**	**+**
β-Phellandrene	555-10-2	10.872	1027	1028 ^c^	0.89 (0.4–1.2)	**+**?	**−**
Eucalyptol	470-82-6	10.943	1028	1026 ^b^	0.63 (0.38–0.87)	**−**	**−**
Perillen					-	**+**	**−**
trans-α-Ocimene					-	**+**	**−**
(E)-Ocimene	502-99-8	11.457	1047	1048 ^d^	0.93 (0.18–2)	**+**	**−**
γ-Terpinene	99-85-4	11.795	1058	1058 ^c^	11.4 (6.63–16.98)	**+**	**−**
cis-Sabinene hydrate	17699-16-0	12.021	1066	1065 ^b^	3.87 (1.59–6.04)	**−**	**−**
trans-Linalool oxide					-	**+**	**−**
cis-Linalool oxide	5989-33-3	12.177	1069	1067 ^b^	0.31 (0–0.39)	**−**	**−**
α-Terpinolene	586-62-9	12.653	1088	1086 ^b^	3.58 (2.92–4.25)	**−**	**−**
trans-Sabinene hydrate	15826-82-1	12.950	1098	1098 ^b^	2.78 (0.64–6.68)	**−**	**−**
Linalool	78-70-6	13.026	1101	1100 ^c^	4.74 (1.55–12.24)	**+**	**+**
cis-p-Menth-2-en-1-ol	29803-82-5	13.630	1122	1121 ^c^	1.08 (0.78–1.51)	**−**	**−**
trans-p-Menth-2-en-1-ol	29803-81-4	14.150	1140	1141 ^c^	0.34 (0.21–0.41)	**−**	**−**
Terpinen-4-ol	562-74-3	15.336	1176	1174 ^b^	19.6 (7.12–36.74)	**+**	**−**
trans-p-Mentha-1(7),8-dien-2-ol	21391-84-4	15.492	1186	1187 ^b^	0.2 (0–0.26)	**−**	**−**
α-Terpineol	98-55-5	15.650	1192	1191 ^c^	1.44 (0.64–2.64)	**−**	**−**
β-Terpineol		10.72				**−**	**+**
cis-Piperitol	34350-53-3	15.805	1197	1195 ^b^	0.17 (0–0.22)	**−**	**−**
Verbenone	80-57-9	16.192	1211	1210 ^c^	0.31 (0–0.38)	**−**	**−**
β-Cyclocitral	432-25-7	16.506	1222	1220 ^c^	0.47 (0.21–0.53)	**−**	**−**
**Sesquiterpenes**					**1.34 (0–2.57)**		
α-Ylangene	14912-44-8	20.632	1375	1373 ^d^	0.4 (0–0.46)	**−**	**−**
a-Copaene	3856-25-5	20.750	1378	1376 ^d^	0.32 (0–0.36)	**−**	**−**
Seychellene	20085-93-2	22.481	1447	1444 ^b^	0.12 (0–0.16)	**−**	**−**
α-Amorphene	20085-19-2	23.381	1485	1483 ^b^	0.52 (0–0.65)	**−**	**−**
α-Farnesene	502-61-4	23.996	1511	1510 ^c^	0.68 (0–1.69)	**+**	**−**
α-Bergamotene					-	**+**	**−**
β-Farnesene					-	**+**	**−**
Farnesane						**−**	**+**
Sesquiterpenoide C_15_H_26_O	-	26.953	1638	-	0.26 (0–0.382)	**−**	**−**
Sesquiterpenoide C_15_H_26_O	-	27.390	1658	-	0.16 (0–0.18)	**−**	**−**
Sesquiterpenoide C_15_H_26_O	-	27.450	1660	-	0.22 (0–0.27)	**−**	**−**
**Aliphatic acids**					**1.3 (0–3.48)**		
Acetic acid	64-19-7	2.261	612	610 ^a^	0.35 (0–0.48)	**−**	**−**
Hexanoic acid	142-62-1	9.640	983	981 ^a^	0.2 (0–0.27)	**−**	**−**
Dodecanoic acid	143-07-7	25.258	1564	1565 ^b^	0.31 (0–0.4)	**−**	**−**
Tetradecanoic acid	544-63-8	29.697	1764	1765 ^a^	2.21 (0–3.08)	**−**	**−**
**Aliphatic ketones**					**0.42 (0–0.96)**		
2,3-Butanedione	431-03-8	2.082	585	587 ^a^	0.24 (0–0.35)	**−**	**−**
1-Penten-3-one	1629-58-9	2.909	676	678 ^a^	0.11 (0–0.12)	**−**	**−**
Acetoin	513-86-0	3.253	706	706 ^a^	0.2 (0–0.24)	**−**	**−**
2-Methyl-3-octanone	923-28-4	9.565	982	984 ^a^	0.26 (0–0.38)	**−**	**−**
**Aliphatic alhehydes**					**9.04 (3.03–15.72)**		
Isobutanal	78-84-2	1.935	554	552 ^a^	0.51 (0.1–1.09)	**−**	**−**
Isopentanal	590-86-3	2.575	643	642 ^a^	1.21 (tr-3.1)	**−**	**−**
2-Methylbutanal	96-17-3	2.671	653	652 ^a^	0.72 (tr-1.35)	**−**	**−**
Pentanal	110-62-3	3.050	700	702 ^b^	0.4 (0.27–0.64)	**−**	**−**
(E)-2-Pentenal	1576-87-0	3.897	745	746 ^a^	0.22 (tr-0.35)	**−**	**−**
Hexanal	66-25-1	4.727	799	801 ^c^	1.23 (0.68–2.25)	**−**	**−**
(E)-2-Hexenal	6728-26-3	5.944	844	846 ^b^	0.37 (0–0.31)	**−**	**−**
Heptanal	111-71-7	7.192	899	901 ^b^	0.32 (tr-0.63)	**−**	**−**
(E)-2-Heptenal	18829-55-5	8.732	949	947 ^b^	0.43 (tr-0.79)	**−**	**−**
(E.E)-2,4-Heptadienal	4313-03-5	9.918	993	994 ^a^	0.25 (0–0.32)	**−**	**−**
Octanal	124-13-0	10.099	1000	998 ^b^	1.1 (0.25–2.17)	**−**	**−**
Nonanal	124-19-6	13.124	1104	1105 ^c^	2.31 (0.62–4.01)	**−**	**−**
(E)-2-Nonenal	2463-53-8	14.721	1159	1157 ^b^	0.26 (0–0.32)	**−**	**−**
Decanal	112-31-2	16.047	1203	1201 ^b^	0.21 (0.12–0.37)	**−**	**+**
(E.E)-2,4-Nonadienal	5910-87-2	16.277	1214	1214 ^c^	0.14 (0–0.13)	**−**	**−**
(E.Z)-2,4-Decadienal	25152-83-4	18.488	1294	1292 ^b^	0.1 (0–0.1)	**−**	**−**
Undecanal	112-44-7	18.857	1307	1305 ^b^	0.19 (0–0.24)	**−**	**−**
(E.E)-2,4-Decadienal	25152-84-5	19.147	1317	1315 ^b^	0.16 (0–0.16)	**−**	**−**
Dodecanal	112-54-9	21.513	1410	1409 ^c^	0.13 (0–0.15)	**−**	**−**
**5,9,13-Trimethyl-4,8,12-tetradecenal**		23.01				**−**	**+**
Tetradecanal	124-25-4	26.404	1613	1613 ^c^	0.18 (0–0.26)	**−**	**−**
Pentadecanal	2765-11-9	28.669	1716	1715 ^a^	0.15 (0–0.27)	**−**	**−**
**Aliphatic alcohols**					**12.8 (4.6–23.68)**		
Methanol	67-56-1	1.532	380	381 ^a^	3.92 (1.4–7.83)	**−**	**−**
Ethanol	64-17-5	1.626	444	445 ^a^	4.96 (0.26–9.85)	**−**	**−**
1-Penten-3-ol	616-25-1	2.852	671	673 ^a^	0.32 (0.12–0.56)	**−**	**−**
Isopentanol	123-51-3	3.548	724	726 ^c^	0.46 (0.14–1.23)	**−**	**−**
1-Pentanol	71-41-0	4.098	757	759 ^c^	0.22 (tr-0.1)	**−**	**−**
(Z)-2-Penten-1-ol	1576-95-0	4.168	763	765 ^b^	0.35 (tr-0.62)	**−**	**−**
(Z)-3-Hexen-1-ol	928-96-1	6.018	848	850 ^b^	0.32 (0.1–0.58)	**−**	**−**
1-Hexanol	111-27-3	6.349	861	863 ^b^	0.84 (0–1.81)	**−**	**−**
(Z)-2-Hepten-1-ol	55454-22-3	9.088	962	N/A	0.12 (0–0.13)	**−**	**−**
1-Heptanol	111-70-6	9.131	966	968 ^c^	0.35 (0–0.42)	**−**	**−**
1-Octen-3-ol	3391-86-4	9.403	974	974 ^b^	0.31 (tr-0.57)	**−**	**−**
1-Octanol	111-87-5	12.114	1069	1070 ^c^	1.15 (0.51–3.02)	**−**	**−**
1-Nonanol	143-08-8	15.082	1172	1172 ^c^	0.16 (0–0.25)	**−**	**−**
1-Hexadecanol	36653-82-4	32.093	1879	1877 ^c^	0.61 (0–0.78)	**−**	**−**
**Alkanes and Alkenes**					**6.19 (3.15–9.67)**		
n-Hexane	110-54-3	2.136	600	600 ^a^	0.25 (0–0.36)	**−**	**−**
(E)-4,8-Dimethyl-1,3,7-nonatriene	19945-61-0	13.479	1117	1117 ^c^	1.31 (0.26–4.58)	**−**	**−**
n-Tridecane	629-50-5	18.654	1300	1300 ^a^	0.21 (0–0.22)	**−**	**+**
n-Tetradecane	629-59-4	21.269	1400	1400 ^a^	0.19 (0–0.19)	**−**	**+ (Tetradecane)**
Pentadecane		18.14				**−**	**+**
n-Hexadecane	544-76-3	26.107	1600	1600 ^a^	0.19 (0–0.3)	**−**	**+ (Hexadecane)**
Heptadecane		21.06				**−**	**+**
Octadecane		22.41				**−**	**+**
Nonadecane		23.70				**−**	**+**
Eicosane		24.93				**−**	**+**
n-Heneicosane	629-94-7	36.269	2100	2100 ^a^	0.86 (0.35–1.91)	**−**	**+ (Heinecosane)**
n-Docosane	629-97-0	38.042	2200	2200 ^a^	0.61 (0.13–1.25)	**−**	**+**
(Z)-9-Tricosene	27519-02-4	39.303	2272	2270 ^d^	0.67 (0.22–1.65)	**−**	**+**
(E)-9-Tricosene	74685-29-3	39.355	2276	N/A	0.97 (0.39–1.79)	**−**	**−**
n-Tricosane	638-67-5	39.744	2300	2300 ^a^	1.69 (0.9–2.33)	**−**	**+**
**Aromatic compounds**					**5.3 (2.09–11.18)**		
Benzaldehyde	100-52-7	8.846	953	952 ^b^	1.26 (0.52–2.9)	**−**	**−**
Benzeneacetaldehyde	122-78-1	11.309	1042	1043 ^d^	2.93 (1.04–8.35)	**−**	**−**
2,2,4,6,6-Pentamethylhept-3-ene		9.31				**−**	**+**
2,6-di-t-butylbenzoquinone		17.79				**−**	**+**
2-Phenylethanol	60-12-8	13.382	1113	1112 ^c^	1.05 (0.52–1.61)	**−**	**−**
1-Methoxy-2-vinylbenzene	612-15-7	14.526	1153	N/A	0.18 (0–0.22)	**−**	**−**
**Furan derivatives**					**0.95 (0.47–1.17)**		
2-Ethyl furan	3208-16-0	3.087	702	704 ^b^	0.11 (0–0.11)	**−**	**−**
2-Pentylfuran	3777-69-3	9.772	986	984 ^b^	0.18 (0.36–1.17)	**−**	**−**
(E)-Dendrolasin	23262-34-2	25.568	1577	1575 ^c^	0.12 (0–0.12)	**+**	**−**
**Other compounds**					**1.54 (0.69–2.3)**		
Hexadecanioic acid					-	**+**	**−**
3-Methylthiopropanal	3268-49-3	7.335	897	901 ^b^	0.74 (tr-1.19)	**−**	**−**
(E)-α-Ionone	127-41-3	22.032	1430	1428 ^b^	0.11 (0–0.12)	**−**	**−**
Birkenal	N/A**	22.360	1444	1443 ^c^	0.2 (0–0.26)	**−**	**−**
(E)-β-Ionone	79-77-6	23.489	1489	1487 ^b^	0.47 (0.26–0.99)	**−**	**−**
Isopropyl tetradecanoate	110-27-0	31.011	1827	1828 ^b^	0.32 (0.11–0.72)	**−**	**−**
**Unidentified compound**					**0.36 (0–0.5)**		
Unidentified compound	-	13.261	1109	-	0.36 (0–0.5)	**−**	**−**

## Data Availability

The original contributions presented in this study are included in the article and Supplementary Material. Further inquiries can be directed to the corresponding author.

## Supplementary Materials

The following supporting information can be downloaded at: https://www.mdpi.com/article/10.3390/plants14060942/s1, Table S1: Data on the pollination systems and the floral volatile compounds (collecting methodology, number of compounds) of *Neottia ovata* and 22 compared orchids; Table S2: The proportion of chemical compounds that appeared only once within the scent profiles of analyzed orchid species. References [17,26,28,29,32,34,58,71,79,80,82,83,84,85,86,87,88,89,90,91,92,93] are cited in the Supplementary Materials.
