# Supplementary material for: Complex Floral Scent Profile of Neottia ovata (Orchidaceae): General Attractants and Beyond
Source: Plants (Basel). 2025 Mar 17;14(6):942. doi: 10.3390/plants14060942 (PMC11946450; doi:10.3390/plants14060942)
Supplement: Supplementary file 1 [file plants-14-00942-s001.zip › plants-3435515-supplementary/Table S1.pdf]

Complex Floral Scent Profile of *Neottia ovata* (Orchidaceae): General Attractants and Beyond

Supplements

Table. S1. Data on the pollination systems and the floral volatile compounds (collecting methodology, number of compounds) of *Neottia ovata* and 22 compared orchids. *Pollination system*: G – generalized, S – specialized, RW – rewarding, FD – food-deception; *group of flower-visiting insects/pollinators*: H –Hymenoptera, D – Diptera, C – Coleoptera, L – Lepidoptera; *the flower volatiles collection methods*: SDE – the combined distillation - elution method, DHS-AE – dynamic headspace sampling with the adsorption-elution method, TD – thermal desorption method, HS-SPME – headspace sampling with solid phase micro-extraction; ? – uncertain data or lack of data.

**Species**	**Pollination system**	**Group of flower-visiting insects/pollinators**	**The flower volatiles collection methods**	**Number of chemical compounds found in flower scent**	**References to:** *visiting insects/pollinators #flower volatiles
Neottia ovata	G, RW	H, D, C/ichneumonids	HS SPME	100	Nillson 1981*#; Presented data#
Disa fragrens	G, RW	H, D, C/?	DHS-AE (hexane:acetone)	48	Johnson and Hobbhahn 2010 *#
Goodyera macrophylla	G, RW	H, D/ bumblebees, syrphids	DHS-AE + SDE (*n*-pentane, diethyl ether)	29	Fernandes et al. 2007 *#
Dactylorhiza viridis	G, RW	H/ichneumonids, ants	HS SPME	11	Claessens and Kleynen 2011 *; Mecca et al. 2022 #
Schizochilus flexuosus	G?, RW	H, L/ bees, wasps	DHS-AE	25	Van der Niet et al. 2010 *#
Gennaria diphylla	S, RW	L/moths	DHS-AE + SDE (*n*-pentane, diethyl ether)	32	Fernandes et al. 2007 *# Claessens et al. 2022 #
Gymnadenia conopsea	S, RW	L/moths	DHS-AE (hexane:acetone)	54	Keiser 1993 #; Huber et al. 2005 #; Claessens and Kleynen 2011*
Gymnadenia nigra	S, RW	L/moths	DHS-AE	47	Keiser 1993 #; Claessens and Kleynen 2011 *
Platanthera bifolia	S, RW	L/moths	DHS-AE	46	Keiser 1993 #; Claessens and Kleynen 2011 *
Platanthera obtusata	S, RW	D/mosquitos	DHS-AE (hexane)	34	Lahondère et al. 2020 *#
Anacamptis coriophora	S, RW	H/ bumblebees, bees	DHS-AE (hexane:acetone)	34	Salzmann et al. 2007 *#

Satyrium longicauda (two ecotypes)	S, RW	L, H/moths, oil collecting bees	DHS-TD	78	Castañeda-Zárate et al. 2021 *#
Himantoglossum hircinum	S, RW?	H/bees	HS SPME	21	Mecca et al. 2021 # Claessens and Kleynen 2011 *
Orchis anthropophora	G, FD	C, H/beetles, ichneumonids	DHS-AE (dichloromethane)	21	Bournéries and Prat 2005 *; Schatz 2006 *; Schatz et al. 2010 #; Claessens and Kleynen 2011 *
Cypripedium calceolus	G/ FD	H,D/bees	DHS-TD	75	Claessens and Kleynen 2011 *, Braunschmid et al. 2021 #
Cypripedium plectrochilum	G, FD	H, D, C, L/bees	HS + TD	7	Li et al. 2008 *#
Himantoglossum robertianum	G, FD	H,C/bees, beetles	HS SPME	70	Romano et al. 2022 *#
Traunsteinera globosa	G, FD	D,H, L, C/flies	DHS-AE (acetone)	41	Claessens and Kleynen 2011 *; Jersáková et al. 2016 #
Neotinea ustulata	S, FD	D,H,C/tachinid fly	DHS-AE	46	Martel et al. 2021 *#
Anacamptis morio	S, FD	H/ bumblebees, bees	DHS-AE (hexane:acetone)	54	Claessens and Kleynen 2011 *; Salzmann et al. 2007 #
Dactylorhiza romana	S, FD	H/ bumblebees, bees	DHS-AE (hexane:acetone)	23	Salzmann and Schiestl 2007 *#
Orchis purpurea	S, FD	H, D/ bees, flies	SDE	37	Claessens and Kleynen 2011 *; Robustelli della Cuna et al. 2022 #
