# Supplementary material for: Complex Floral Scent Profile of Neottia ovata (Orchidaceae): General Attractants and Beyond
Source: Plants (Basel). 2025 Mar 17;14(6):942. doi: 10.3390/plants14060942 (PMC11946450; doi:10.3390/plants14060942)
Supplement: Supplementary file 1 [file plants-14-00942-s001.zip › plants-3435515-supplementary/Table S2.pdf]

Complex Floral Scent Profile of *Neottia ovata* (Orchidaceae): General Attractants and Beyond

Table S2. The proportion of chemical compounds that appeared only once within the scent profiles of analyzed orchid species (selected for the presented analyses). The single compounds were categorized according to chemical groups and orchid pollination strategy groups.

		Number of single compounds	Per cent
Terpenes	Generalists RW	14	43.75
	Specialists RW	10	40
	Generalists FD	16	53.34
	Specialists FD	8	32
Aliphatic compounds	Generalists RW	6	37.5
	Specialists RW	9	52.94
	Generalists FD	12	63.16
	Specialists FD	4	44.45
Aromatic compounds	Generalists RW	11	45.83
	Specialists RW	17	44.74
	Generalists FD	4	30.77
	Specialists FD	4	25
